# Sleep Duration Modifies the Association of the Alternate Mediterranean Diet Score With Metabolic Syndrome in Midlife Women in Mexico

**DOI:** 10.1016/j.tjnut.2025.08.023

**Published:** 2025-08-29

**Authors:** Haneen Bou Ghanem, Maria M Kofas, Karen E Peterson, Alejandra Cantoral, Abeer Aljahdali, Libni Torres-Olascoaga, Martha M Tellez-Rojo, Erica C Jansen

**Affiliations:** 1 Department of Nutritional Sciences, University of Michigan, Ann Arbor, MI, United States; 2 Department of Health, Iberoamerican University, Mexico City, Mexico; 3 Department of Clinical Nutrition, King Abdulaziz University, Jeddah, Saudi Arabia; 4 Center for Nutrition and Health Research, National Institute of Public Health, Cuernavaca, Mexico

**Keywords:** metabolic syndrome, alternate Mediterranean diet, Mediterranean diet, sleep duration, midlife women

## Abstract

**Background:**

Adherence to a Mediterranean diet and better sleep health have independently been associated with a lower risk of metabolic syndrome (MetS); however, their combined effect has rarely been considered.

**Objectives:**

This study aimed to examine whether sleep duration modifies the association between adherence to the alternate Mediterranean (aMed) diet and MetS among midlife Mexican women.

**Methods:**

The analytic sample consisted of 410 women with a mean age of 48.2 ± 6.1 years participating in the Early Life Exposure in Mexico to ENvironmental Toxicants (ELEMENT) study. Diet was assessed with a validated food frequency questionnaire (FFQ). The mean daily sleep duration was measured with 7-d wrist actigraphy (Actigraph GTX-BT). MetS was defined on the basis of the American Heart Association/National Heart, Lung, and Blood Institute criteria. Multivariable logistic regression models were used to estimate the association between higher adherence to the aMed diet and MetS and between sleep duration and MetS, each controlling for age, socioeconomic status, and moderate-to-vigorous physical activity. To assess the potential modifying role of sleep duration, stratified analysis by sleep status was conducted, where participants were categorized as having adequate sleep (≥7 h/d) or inadequate sleep (<7 h/d) on average across the week. Statistical interaction was also tested in a logistic model.

**Results:**

MetS was identified in 49.8% of participants, and 56.6% had a mean sleep duration of <7 h/d. Neither higher adherence to the aMed diet nor sleep duration alone was associated with MetS. However, the stratified analysis showed that among women with inadequate sleep, higher adherence to the aMed diet was related to 16% lower odds of MetS (odds ratio: 0.84; 95% CI: 0.69, 0.99; *P*-interaction = 0.09).

**Conclusions:**

Associations between diet and MetS are only observed in the presence of inadequate sleep duration, suggesting a potential interaction that warrants further investigation through longitudinal research.

## Introduction

Metabolic syndrome (MetS) is a multifactorial disorder characterized by a cluster of interrelated cardiometabolic risk factors, including central obesity, insulin resistance, dyslipidemia, and hypertension [1,2]. The prevalence of MetS in Mexico is a growing public health concern, increasing from 40.2% in 2006 to 56.31% in 2018, affecting ∼36.5 million adults aged ≥20 years [3,4]. The causes of MetS are likely due to a complex interplay among genetic, environmental, and lifestyle factors, including diet and sleep [2].

One dietary pattern that has been associated with a reduced risk of MetS, particularly among the Mediterranean and European populations, is the Mediterranean diet (MedDiet) due to its anti-inflammatory and cardioprotective properties [[5], [6], [7], [8], [9], [10], [11]]. The MedDiet is characterized by high consumption of fruits, vegetables, whole grains, nuts, and olive oil, with moderate consumption of fish and limited intake of red meat and processed foods. This diet contains high levels of dietary fiber, complex carbohydrates, essential vitamins and minerals, ω-3 (n–3) and ω-9 fatty acids, antioxidants, polyphenols, and other bioactive compounds, which reduce oxidative stress and inflammation, enhance insulin sensitivity, and improve lipid metabolism, contributing to the overall prevention and management of MetS [5,8,12]. The MedDiet was originally developed on the basis of foods and eating practices from the Mediterranean regions, which may not fully align with the cultural preferences of other populations, including Mexico. However, the alternate Mediterranean (aMed) diet aligns with the principles of the MedDiet while taking into account regional variations in food availability and preferences [13], and it has been previously implemented to characterize dietary intake among a Mexico City population [14].

As another important modifiable lifestyle factor, sufficient sleep is increasingly recognized for its role in preventing adverse cardiometabolic health [15,16]. Among the various types of sleep disturbances, the United States Centers for Disease Control and Prevention declared inadequate sleep a public health epidemic, reporting that over one-third of American adults do not get enough sleep regularly, defined as sleeping <7 h per night [15,17]. A meta-analysis of epidemiologic studies involving 634,511 participants found that short sleep duration was linked to higher odds of obesity [16]. Furthermore, a prospective cohort study of 2579 adults found that individuals sleeping <6 h/d had a significantly higher risk of developing MetS, with short sleepers being at a greater risk of having higher waist circumference, blood pressure, fasting glucose, and lower HDL cholesterol [18].

Adherence to the MedDiet and obtaining adequate sleep have been independently associated with a reduced risk of MetS [7,9,18,19]. However, these associations have not been observed consistently across all studies [20]. In addition, the MedDiet has been identified as a predictor of better sleep quality [21]. Yet, the interaction between MedDiet and sleep duration in shaping the MetS risk is still underexplored. Intriguingly, a previous study of Greek adults found that adherence to the MedDiet was related to a 70% lower 20-year cardiovascular disease (CVD) risk only among adequate sleepers (≥7 h) [22]. However, this investigation was limited by reliance on self-reported sleep duration rather than on objective estimates based on actigraphy. This evidence highlights the potential of sleep in modifying the association between the MedDiet and MetS, which is worth replicating in different populations. Thus, this study aimed to examine whether an interaction exists between objectively measured sleep duration and adherence to the aMed diet in relation to MetS in midlife Mexican women.

## Methods

### Study population

The study population was derived from the Early Life Exposure in Mexico to ENvironmental Toxicants (ELEMENT) cohort study, which was established in the mid-1990s. ELEMENT includes 3 birth cohorts comprising 1643 mother–child pairs sequentially recruited between 1994 and 2003 during pregnancy or after delivery from family clinics in Mexico City, belonging to the Instituto Mexicano del Seguro Social [23]. The study population for this analysis included women from the 2019–2022 follow-up visit, which encompassed 587 mothers who were originally recruited in ELEMENT. The first set of data (*n* = 217) was collected between 2019 and 2020, before the COVID-19 pandemic. Data collection resumed from 2021 to 2022 and included the remaining 370 women. A total of 410 participants who had ≥4 nights of processable sleep data collected via wrist actigraphy over 1 week were included in this study. The recruitment process and subsequent follow-up of participants in the ELEMENT study have been previously described [23].

### aMed diet score

Dietary intake was assessed using a semiquantitative food frequency questionnaire (FFQ) validated in the Mexican population [24]. The FFQ queries the frequency of consumption and usual serving sizes for 140 foods and beverages consumed during the 7 days preceding the interview. The frequency of consumption of each food and drink listed in the FFQ was multiplied by the portion size (in grams or milliliters), the number of portions, and the number of days, and then divided by 7 to assess the mean daily intake. Further details on the computation of the dietary data are provided in a previous publication [25].

We used the aMed diet score in this study, which assessed adherence to the MedDiet in populations where some traditional Mediterranean foods may not be commonly consumed, including the Mexican population. The aMed diet score was adapted from the work of Trichopoulou et al. [26] on the MedDiet score in Greece by Fung et al. [13]. The aMed score includes 9 categories: vegetables (excluding potatoes), legumes, fruits, nuts, whole grains, red and processed meat, fish, MUFAs-to-SFAs ratio, and alcohol. To calculate the aMed score, a score of 1 is assigned to each healthy food category when the mean intake exceeds the median intake. Similarly, a score of 1 is given when the mean intake falls below the median intake for unhealthy components [13]. In this study, we excluded alcohol because our study population had very limited alcohol intake. Therefore, the computed aMed scores range from 0 to 8.

### Sleep duration

To assess the mean daily sleep duration and moderate-to-vigorous physical activity (MVPA), participants were given wrist actigraphy devices (Actigraph GTX-BT) to wear continuously on their nondominant wrists for 7 days. At the same time, participants were provided with sleep diaries to record their bedtime and wake time. The purpose of the sleep diary was to improve the accuracy of the sleep estimation algorithm. Data were collected for 5 weekdays and 2 weekend days for each participant.

Nightly sleep parameters were determined from the actigraphy data, using 60-sec epoch lengths. This analysis used a pruned dynamic programming algorithm developed in R (R Foundation for Statistical Computing) called Actisleep, which has been validated against polysomnography, the gold standard method for sleep assessment [27]. The pruned dynamic programming algorithm was used to compute sleep duration in minutes during weekdays and weekends. However, because the algorithm cannot differentiate between naps and daytime sedentary behavior, naps were not included as one of the assessed sleep parameters. A weighted mean of minutes per day was computed to determine the average nightly sleep duration for the study participants. Weekday sleep duration was weighted by a factor of 5, and weekend sleep duration by a factor of 2 before dividing the total amount of sleep by 7 to obtain the overall weekly mean of sleep per day. Women who slept, on average, <7 h/d were classified into the inadequate sleep group, and women who slept for ≥7 h/d were assigned to the adequate sleep group. Various thresholds have been used in the literature to define short sleep duration. We chose the 7-h threshold based on the United States Centers for Disease Control and Prevention and the American Academy of Sleep Medicine definition of short sleep duration [17,28].

### Metabolic syndrome

The MetS variable was computed according to the American Heart Association/National Heart, Lung, and Blood Institute criteria. Women were diagnosed with MetS if they exhibited 3 or more of the following conditions, measured at the in-person visit using standardized protocols: waist circumference > 35 inches, fasting triglycerides ≥ 150 mg/dL or on drug treatment for elevated triglycerides, HDL cholesterol < 50 mg/dL or taking medication for reduced HDL cholesterol, blood pressure ≥ 130/85 mmHg or on drug treatment for elevated blood pressure, and fasting glucose ≥100 mg/dL or on drug treatment for elevated glucose concentrations [2].

### Covariates

Several covariates were considered potential confounders due to their association with either the exposure or the outcome, including age, Body Mass Index (BMI), socioeconomic status (SES), smoking status, menopausal status, and physical activity [[29], [30], [31]]. Data on these covariates were obtained through an interviewer-administered questionnaire on sociodemographic characteristics and health behaviors, along with anthropometric measurements conducted during the visit. The age of participants, represented in years, was treated as a continuous variable. BMI (in kg/m^2^) was derived from height and weight measurements obtained by trained research personnel following standardized protocols [32]. SES was evaluated using a categorical index developed by the Mexican Association of Market Research Agencies (Asociación Mexicana de Agencias de Investigación de Mercado y Opinión Pública), which incorporates both income and educational attainment into 7 socioeconomic levels (A/B, C+, C, C-, D+, D, and E). This index was then condensed into 3 categories: low (E, D, and D+), medium (C-, C, and C+), and high (A/B) SES [33]. Smoking behavior was assessed using the Adult Health Behavior module derived from the National Health Interview Survey. Participants’ smoking status was categorized into 3 groups: current smokers, former smokers, and nonsmokers [34]. The menopause status was self-reported and classified into 2 categories: premenopause/perimenopause and postmenopause, determined by the self-reported timing of the last menstrual cycle, with 12 months as the defining cutoff.

To estimate the MVPA from the Actigraphy data, 5-s epochs from the nonsleep period were utilized as input for GGIR, an R package used to analyze multiday raw accelerometer data for physical activity and sleep research [35]. The cutoff values for the duration of daily light, moderate, and vigorous-intensity physical activity used were 45, 101, and 429 min, respectively, based on relevant validation studies [35,36]. We summed the daily MVPA and then computed a weighted mean per minute per day.

### Statistical analysis

Continuous variables were characterized by their means and standard deviation (SD), and categorical variables by their frequencies and proportions. Log transformation was applied for continuous variables exhibiting a skewed distribution, such as BMI and all the food categories of the aMed score. All food groups in the aMed score were adjusted for energy using the residual method [37]. Bivariate analyses were conducted to explore the relationships between potential covariates with the exposures, sleep duration and the aMed diet, and the outcome, MetS. Continuous variables were analyzed using analysis of variance or *t* tests for normally distributed data and Wilcoxon rank-sum tests for non-normally distributed data. Categorical variables were compared using the χ^2^ tests. Multivariable regression models were used to assess the impact of higher adherence to the aMed diet on MetS while controlling for relevant covariates, including age, SES, and MVPA. These covariates were selected on the basis of a priori knowledge of important confounders and for showing associations with sleep and/or the aMed diet in bivariate analyses (not necessarily statistically significant). Additionally, we examined the association between sleep duration and MetS, adjusting for the same covariates. The mean sleep duration per day was also examined as a modifier in the relationship between the aMed score and MetS, where the full cohort was divided into 2 groups based on their sleep duration. Multivariable regression analysis was performed separately for each subgroup, adjusting for the same covariates as in the full model. To statistically test for the significance of the effect modification, we tested for the interaction between sleep duration and the aMed diet in relation to MetS. Given that interaction analyses are underpowered, the interaction was considered significant if the *P* value was ≤0.10. However, for all other results, statistical significance was defined as a *P* value of <0.05.

As part of the sensitivity analysis, menopause status was included as a covariate in the fully adjusted models. We also conducted another sensitivity analysis to assess whether the date of the data collection, pre-COVID compared with post-COVID visits (2019–2020 and 2021–2023, respectively), impacted the results. The analyses were performed using the SAS statistical software package, version 9.4, and R, version 4.3.0 (R Foundation for Statistical Computing).

## Results

The study included 410 women with a mean age of 48.2 ± 6.1 years and a BMI of 29.6 ± 4.9 kg/m². The overall prevalence of MetS in midlife Mexican women was 49.8%. Table 1 presents the descriptive statistics of all the study participants based on their adherence to the aMed diet. There was a positive association between aMed score and age. Similarly, postmenopausal women were more likely to be in the highest quartile of the aMed diet score than premenopausal and peri-menopausal women. In addition, women with higher adherence to the aMed diet tended to have higher SES.

**TABLE 1:** Characteristics of participants by quartiles of the aMed score (*n* = 410).

Variable^1^			aMed diet score				*P*
			Q1 Median = 2	Q2 Median = 3	Q3 Median = 4	Q4 Median = 5	
		Mean (SD)					

Age (y)		48.2 (6.1)	46.4 (5.8)	48.4 (7.3)	48.9 (5.2)	49.5 (6.1)	<0.001
BMI (kg/m^2^)		29.6 (4.9)	29.7 (5.0)	29.5 (4.8)	29.8 (5.1)	29.3 (4.9)	0.63

		*n* (%)					

SES, based on AMAI classification^2^	High SES	42 (10.3)	10 (23.8)	4 (9.5)	19 (45.2)	9 (21.4)	0.06
	Medium SES	308 (75.7)	91 (29.5)	64 (20.8)	76 (24.7)	77 (25)	
	Low SES	57 (14)	18 (31.6)	16 (28.1)	13 (22.8)	10 (17.5)	
Smoking	Current smoker	107 (26.2)	36 (33.6)	23 (21.5)	27 (25.2)	21 (19.6)	0.64
	Former smoker	68 (16.7)	19 (27.9)	15 (22.1)	21 (30.9)	13 (19.1)	
	Non-smoker	233 (57.1)	63 (27)	47 (20.2)	60 (25.7)	63 (27.04)	
Menopausal status	Premenopause/perimenopause	217 (53.8)	76 (35)	41 (18.9)	58 (26.7)	42 (19.3)	0.01
	Postmenopause	186 (46.1)	41 (22)	42 (22.6)	49 (26.3)	54 (29)	
MetS	No	206 (50.2)	55 (26.70)	43 (20.9)	55 (26.7)	53 (25.7)	0.68
	Yes	204 (49.8)	64 (31.4)	42 (20.6)	54 (26.5)	44 (21.6)	
		Mean (SD)					

Sleep per day (min)		407.1 (70.2)	409.8 (64.6)	402.5 (65.4)	407.5 (79.4)	407 (70.6)	0.85
MVPA per day (min)		112.6 (47.5)	109.7 (48.4)	116.6 (48.4)	112.9 (51.2)	112.4 (41.6)	0.76
Dietary intake							
Energy intake (kcal)		1883 (668)	1868 (622)	1787 (568)	1855 (716)	2018 (734)	0.20
Fruits (g)		332.2 (244.2)	251 (193)	256 (230)	362 (239)	465 (256)	<0.001
Vegetables (g)		294.4 (216.1)	187 (122)	229 (149)	351 (245)	421 (236)	<0.001
Whole grains (g)		107.7 (67.5)	93.7 (66.5)	112 (71.9)	113 (66.5)	116 (64.4)	<0.001
Legumes (g)		26.3 (23.0)	16.7 (13.3)	24.2 (18.9)	30.4 (26.2)	35.2 (27)	<0.001
Fish (g)		14.0 (22.5)	4.2 (7.1)	10 (15)	19.5 (30.5)	23.2 (24.5)	<0.001
Meat (g)		42.0 (38.7)	57 (44.8)	36.3 (32.1)	42.7 (43)	28 (20.7)	<0.001
Nuts (g)		5.9 (10.3)	3 (6.1)	3.24 (6.8)	5.85 (9.44)	11.8 (14.7)	<0.001

Abbreviations: aMed, alternate Mediterranean; MetS, metabolic syndrome; MVPA, moderate-to-vigorous physical activity; SES, socioeconomic status.

1 Some variables have missing data, as follows: AMAI (*n* = 3), smoking (*n* = 2), and menopausal status (*n* = 7).

2 AMAI: Asociación Mexicana de Agencias de Investigación de Mercado y Opinión Pública (Mexican Association of Market Research Agencies).

More than half (56.6%) of the women had inadequate sleep, defined as sleeping <7 h/d. Women in the adequate sleep group (*n* = 178) had a mean of 470.8 ± 42.1 min (7.8 ± 0.7 h) of sleep per day, whereas those in the inadequate sleep group (*n* = 232) had 358.1 ± 42.7 min (6.0 ± 0.7 h) of sleep per day (Table 2). No significant variations were observed in age, BMI, smoking status, menopausal status, MetS prevalence, or adherence to the aMed diet between the two groups. However, women in the inadequate sleep group had significantly higher levels of MVPA per day (119.5 ± 45 min) than those in the adequate sleep group (103.6 ± 49.4 min; *P* ≤ 0.001).

**TABLE 2:** Characteristics of participants by adequate and inadequate sleep groups.

Variable^1^		Adequate sleep group (*n* = 178)	Inadequate sleep group (*n* = 232)	*P*
		Mean (SD)		

Age (y)		48.5 (6.5)	48.0 (5.8)	0.37
BMI (kg/m^2^)		29.4 (5.0)	29.8 (4.9)	0.44

		*n* (%)		

SES, based on AMAI classification^2^	High SES	17 (9.7)	25 (10.8)	0.10
	Medium SES	126 (72)	182 (78.4)	
	Low SES	32 (18.3)	25 (10.8)	
Smoking	Current smoker	40 (22.6)	67 (29.0)	0.24
	Former smoker	34 (19.2)	34 (14.7)	
	Non-smoker	103 (58.2)	130 (56.3)	
Menopausal status	Premenopause/perimenopause	89 (50.9)	128 (56.1)	0.30
	Postmenopause	86 (49.1)	100 (43.9)	
MetS	No	86 (48.3)	120 (51.7)	0.49
	Yes	92 (51.7)	112 (48.3)	
		Mean (SD)		

aMed diet score		3.5 (1.5)	3.4 (1.6)	0.40
Sleep duration per day (min)		470.8 (42.1)	358.1 (42.7)	<0.0001
MVPA per day (min)		103.6 (49.4)	119.5 (45)	0.01

Abbreviations: aMEd, alternate Mediterranean; MetS, metabolic syndrome; MVPA, moderate-to-vigorous physical activity; SES, socioeconomic status.

1 Some variables have missing data, as follows: AMAI (*n* = 3), smoking (*n* = 2), and menopausal status (*n* = 7).

2 AMAI: Asociación Mexicana de Agencias de Investigación de Mercado y Opinión Pública (Mexican Association of Market Research Agencies).

Table 3 shows the associations among the aMed Diet score, sleep duration, and MetS. In the full-cohort analysis, no significant association was found between adherence to the aMed diet and MetS, with an odds ratio (OR) of 0.95 (95% CI: 0.83, 1.08). Moreover, sleep duration was not associated with the odds of MetS, whether modeled categorically or continuously (per-hour increase; OR: 1.00; 95% CI: 0.84, 1.20). However, there was evidence of an interaction between the aMed diet and sleep duration, such that there was a positive association between higher adherence to the aMed diet and lower odds of MetS only among the inadequate sleepers (*P*-interaction = 0.09). Stratified analysis showed that among the women in the inadequate sleep group, the odds of having MetS were 16% lower for women with higher adherence to the aMed diet (95% CI: 0.69, 0.99) than those with lower adherence. However, in the adequate sleep group, higher adherence to the aMed diet was not significantly associated with MetS (OR: 1.11; 95% CI: 0.90, 1.37). Sensitivity analyses indicated that including menopause status as a covariate in the fully adjusted models did not alter the finding. Similarly, the results did not differ significantly when accounting for the timing of data collection (pre-COVID compared with post-COVID visits: 2019–2020 compared with 2021–2023).

**TABLE 3:** Associations of aMed diet score and sleep duration with MetS

Exposure	Unadjusted OR (95% CI)	Adjusted OR (95% CI)^1^	*P*
aMed diet score			
Quartile 1	1.29 (0.74, 2.24)	1.30 (0.73, 2.30)	0.37
Quartile 2	1.10 (0.64, 1.91)	1.10 (0.62, 1.95)	0.74
Quartile 3	Reference	Reference	
Quartile 4	0.92 (0.53, 1.60)	0.88 (0.50, 1.54)	0.65
Continuous, per point	0.96 (0.85, 1.10)	0.95 (0.83, 1.08)	0.47
Sleep duration			
Quartile 1, 2.9 to <6.0 h	0.98 (0.57, 1.70)	0.92 (0.52, 1.61)	0.76
Quartile 2, 6.0 to <6.8 h	0.70 (0.38, 1.15)	0.62 (0.35, 1.10)	0.10
Quartile 3, 6.8 to <7.6 h	Reference	Reference	
Quartile 4, 7.6 to 10.2 h	1.08 (0.62, 1.88)	0.78 (0.43, 1.39)	0.40
Continuous, per hour	1.08 (0.91, 1.27)	1.00 (0.84, 1.20)	0.95

Abbreviations: aMed, alternate Mediterranean; MetS, metabolic syndrome; OR, odds ratio.

1 Adjusted for age, socioeconomic status, and moderate-to-vigorous physical activity. Food groups were adjusted for energy before computing the aMed score.

## Discussion

Within this sample of midlife Mexican women, a higher aMed diet score was linked to 16% lower odds of having MetS only among midlife women who slept <7 h/d. Neither the aMed diet nor sleep duration alone was associated with MetS in this population. Altogether, these findings highlight possible effect modification between diet adherence and sleep health in relation to MetS among women.

The presence of effect modification by sleep duration status on the MedDiet and cardiometabolic outcomes is supported by a few studies, although they were not specifically conducted among midlife women. The ATTICA cohort study, which followed up 313 Greek adults for 20 years, reported an interaction between the MedDiet and sleep in relation to CVD risk. Yet, rather than noting an association among the inadequate sleepers, the ATTICA study found that higher adherence to the MedDiet was inversely associated with CVD risk among individuals with adequate sleep habits (≥7 h/d), with a 70% reduction in 20-y CVD risk observed in this group [22]. A study among 23,212 participants from the NHANES reported an interaction between the MedDiet and sleep disorders on CVD-related mortality (*P* = 0.033). Participants with higher adherence to the aMed diet who also had sleep disorders had a synergistically lower risk of all-cause and CVD-related mortality [38]. Collectively, the previous studies and ours highlight the possibility of sleep and diet interactions with respect to cardiometabolic outcomes, previously discussed as an underexplored area in investigations of sleep, diet, and cardiometabolic health [39].

Our finding that the aMed diet showed an inverse association with MetS only among the short sleepers highlights a possible synergistic effect between these two lifestyle factors. Sleep deprivation is known to dysregulate metabolic pathways, including glucose homeostasis, which directly impacts how food is metabolized [40]. In this state, the availability of a high-quality diet, such as the aMed diet, may become even more relevant. Moreover, longer-term sleep deprivation results in higher inflammation [41] and increased blood pressure [18], and the MedDiet is known for its anti-inflammatory and antihypertensive effects. For example, the frequent consumption of olive oil and fish in a Mediterranean dietary pattern is associated with a higher intake of beneficial fatty acids, including ω-3 and ω-9 fatty acids, which also have antioxidant and anti-inflammatory properties [36]. Oleic acid in olive oil can also manage blood pressure by inhibiting the angiotensin-converting enzyme pathway [36].

Contrary to expectations [7,9,10], there were null associations between the aMed score and MetS in the overall sample. One plausible explanation is reverse causation bias, which could be especially noticeable in our population of middle-aged women. The midlife stage is often characterized by lifestyle changes in which women with metabolic disorders are more inclined to adopt healthier dietary patterns to protect their health. This might explain the significant positive association observed between the aMed score with age and menopausal status. However, it is notable that a longitudinal study of 334 women of Mexican ethnic descent who participated in the Women's Health Initiative similarly found that the aMed diet, along with other diet-quality indices, was not associated with the overall risk of MetS [20].

The null association between sleep duration and MetS was also unexpected and may similarly be due to the cross-sectional nature of the study, which is subject to reverse causation bias. Algorithm errors in the estimation of sleep from the Actigraph data could also be a factor, because distinguishing true sleep from other sedentary behaviors during the sleep period (e.g., lying quietly in bed but awake) can be challenging, especially for populations at risk of insomnia, such as midlife women [42].

This study has several strengths, including the use of actigraphy to assess sleep duration per day, which offered objective and accurate measurements, reducing potential bias associated with self-reported data. We also used a validated FFQ to assess dietary intake, which enhanced the ability to capture true dietary patterns. Although our study provides valuable insights, it is not without limitations. The cross-sectional design precludes causal inferences, and it is also subject to reverse causation bias, where women with MetS might have higher aMed scores due to recent improvements in diet in order to address health conditions. Additionally, although we adjusted for several potential confounders, residual confounding cannot be entirely ruled out. We also used the aMed diet, which may not fully capture key food groups in the traditional Mexican diet. However, it was designed to assess adherence to Mediterranean dietary principles in non-Mediterranean populations by accounting for regional variations in dietary patterns. Finally, we were underpowered to detect a statistically significant interaction at a standard threshold of *P* < 0.05.

Our findings contribute to the growing body of literature on the interactions among diet, lifestyle factors, and metabolic health. The high prevalence of MetS in our cohort highlights the significant metabolic health challenges faced by midlife women in Mexico. In conclusion, these cross-sectional analyses revealed that women with inadequate sleep showed lower odds of MetS when their adherence to the aMed diet was higher. These results suggest that following a healthy diet may be more beneficial for those with insufficient sleep. This work underscores the importance of considering both dietary and sleep habits in future longitudinal and intervention studies related to MetS.

## Author contributions

The authors’ responsibilities were as follows: HBG, MMK, ECJ, KEP, AA: designed the research; LT-O, MMTR: conducted the research; HBG, MMK: analyzed the data; HBG: wrote the paper; MMK, ACP, AA, LT-O, MMTR, KEP, ECJ: provided critical feedback and editorial review; KEP, MMTR: secured funding and resources necessary to conduct the research; and all authors: have read and approved the manuscript.

## Data availability

Data described in the manuscript, code book, and analytic code will be made available upon request to KEP and MMTR, pending review and approval by the ELEMENT Executive Committee.

## Declaration of generative AI and AI-assisted technologies in the writing process

During the preparation of this work, the author(s) used Grammarly to check grammar and rephrase some sentences. After using this tool/service, the author(s) reviewed and edited the content as needed and take(s) full responsibility for the content of the publication.

## Funding

This study was funded in part by 1R01ES032330, R24ES028502, and U24ES028502. ECJ reports NIH funding over the course of this study (K01 HL151673).

## Conflict of interest

KEP reports financial support was provided by National Institute of Environmental Health Sciences. EJC reports financial support was provided by National Heart Lung and Blood Institute. All other authors report no conflicts of interest.
